# Evaluating the effect of action-like video game play and of casual video game play on anxiety in adolescents with elevated anxiety: protocol for a multi-center, parallel group, assessor-blind, randomized controlled trial

**DOI:** 10.1186/s12888-024-05515-7

**Published:** 2024-01-19

**Authors:** Naïma Gradi, Adrien Chopin, Daphné Bavelier, Tomer Shechner, Swann Pichon

**Affiliations:** 1 Department of Psychology, University of Geneva, Geneva, Switzerland; 2 Smith Kettlewell Eye Research Institute, San Francisco, CA USA; 3 Department of Psychology, University of Haifa, Haifa, Israel; 4 Geneva School of Health Sciences, Geneva, Switzerland

**Keywords:** Anxiety, Adolescent, Video games, Randomized controlled trial, Digital mental health, Cognitive training, Emotion regulation, Online intervention

## Abstract

**Background:**

Adolescence is a critical period for the onset and maintenance of anxiety disorders, which raises the importance of intervening early; one possibility of doing so is via digital interventions. Within that research field, at least two important research paths have been explored in the past years. On the one hand, the anxiolytic effect of casual video games has been tested as such gaming activity may distract away from anxious thoughts through the induction of flow and redirection of attention toward the game and thus away of anxious thoughts. On the other hand, the bidirectional link between weak attentional control and higher anxiety has led to the design of interventions aiming at improving attentional control such as working memory training studies. Taking stock that another genre of gaming, action video games, improves attentional control, game-based interventions that combines cognitive training and action-like game features would seem relevant. This three-arm randomized controlled trial aims to evaluate the feasibility and the efficacy of two video game interventions to document how each may potentially alleviate adolescent anxiety-related symptoms when deployed fully on-line.

**Methods:**

The study aims to recruit 150 individuals, 12 to 14 years of age, with high levels of anxiety as reported by the parents’ online form of the Screen for Child Anxiety Related Disorders questionnaire. This trial contrasts a child-friendly, “action-like” video game designed to improve attentional control abilities in a progressive and stepwise manner (Eco-Rescue), a casual puzzle video game selected to act as a positive distraction tool (Bejeweled) and finally a control group with no assigned training intervention to control for possible test-retest effects (No-training). Participants will be assigned randomly to one of the three study arms. They will be assessed for main (anxiety) and secondary outcomes (attentional control, affective working memory) at three time points, before training (T1), one week after the 6-week training (T2) and four months after completing the training (T3).

**Discussion:**

The results will provide evidence for the feasibility and the efficacy of two online video game interventions at improving mental health and emotional well-being in adolescents with high levels of anxiety. This project will contribute unique knowledge to the field, as few studies have examined the effects of video game play in the context of digital mental health interventions for adolescents.

**Trial registration:**

The trial is registered on ClinicalTrials.gov (NCT05923944, June 20, 2023).

## Background

### Anxiety - adolescence

Anxiety is the most frequently diagnosed adolescent mental health problem in children and adolescents, with an estimated prevalence of about 7% [1, 2]. Beyond diagnosed anxiety disorders, Balász et al. [3] observed in their study that 32% of adolescents experienced subclinical levels of anxiety. Anxiety is characterized by emotion dysregulation [4, 5] and behavioural over-control [6]. Anxiety symptoms are associated with a lower general quality of life [7], worse school performance [8] and lower social functioning [9]. Moreover, adolescent anxiety tends to persist into adulthood and is related to other health problems, such as substance misuse and depression [10, 11]. Epidemiological evidence indicates that for some anxiety disorders, 50% of diagnosed adolescents show a first onset before the age of 15 [12]. Adolescence is indeed a transitional period key for structuring socialization and well-being, characterized by a limited efficacy in emotion regulation strategies and in applying attentional control in emotional contexts, as well as heightened levels of anxiety and negative mood. At the same time, adolescence is also a developmental stage highly plastic [13–15] and constitutes a sensitive window of opportunity to benefit from interventions aiming at promoting cognition and emotion regulation.

### Cognitive model of anxiety: attentional control, anxiety and emotion regulation

Emotion regulation refers to the ability to interact with emotions in a way that is consistent with personal goals [16]. Theories of emotion regulation and cognitive theories of anxiety generally agree that the capacity to cognitively regulate emotions relies on the same cognitive functions and prefrontal networks which support attentional control allowing the selection and manipulation of information (such as stimuli or thoughts) in working memory (WM) [17–24].

In the context of anxiety, individuals may have more difficulties inhibiting irrelevant information or anxious thoughts, and may therefore experience higher difficulties to prevent their access to WM. As a result, the WM capacity available for task performance is likely restricted as well as the ability to retain relevant information [25–29]. This hypothesis receives support from meta-analytical findings [29] showing the association between self-report levels of anxiety and poorer performance on measures of WM capacity (g=-0.33), in particular visuospatial WM (g=-0.41).

The underlying assumption of cognitive models of anxiety is that efficient attentional control (AC)– the ability to monitor and flexibly allocate resources to goal-relevant information while inhibiting goal-irrelevant information - is a key component for implementing cognitive emotion regulation strategies and to filter out distracting emotional stimuli or undesired thoughts. This has been backed up by evidence that interindividual differences in AC is linked to more frequent or efficient use of adaptive emotion regulation strategies, such as reappraisal [30–32], as well as neurobiological evidence that brain networks responsible for implementing emotion regulation strategies recruit regions involved in AC. In a reciprocal manner, cognitive theories of anxiety posit that anxiety impairs the efficiency of AC [24, 33], and that this in turn is likely to impact negatively individual’s ability to regulate emotions and anxiety. Previous studies have highlighted the relationship between impairments in AC skills and higher levels of anxiety in children and adolescents [34–36].

### Review of computerized cognitive trainings (CCT) and casual video game interventions

Based on the assumption that deficits in AC contribute to the etiology and maintenance of anxiety and emotion dysregulation, researchers have developed cognitive training interventions designed to train specific cognitive processes with the goal of reducing anxiety symptoms. These interventions, which have been termed computerized cognitive trainings (CCT), may offer a potentially interesting cognitive training remediation tool as well as augment treatment accessibility, since they may be performed at home and do not require the presence of a specialist.

So far, CCT interventions have often targeted the training of WM since reduced WM capacity is often identified as one of the primary cognitive deficits exhibited in individuals with anxiety [29]. Intervention studies targeting WM have commonly used the adaptive dual n-back task with the goal to increase WM resources in anxious individuals [37, 38]. Different versions of n-back training have been used, with the most well-designed ones incorporating adaptive difficulty to account for interindividual differences in executive functions and their potential for plastic changes through training. Some adaptive n-back trainings have included affective stimuli (commonly negative valence materials such as words or pictures) with the goal of reducing excessive processing of task-irrelevant affective stimuli, interfering with on-going task performance both at the perceptual level and at the executive control level. Such prioritization of affective information is proposed to be increased in individuals suffering from anxiety because of hypervigilance towards affective information. Emotional n-back training is suggested to be more prompt to induce greater reduction of anxiety symptoms by targeting affective executive control [39, 40].

A closely related field of research has developed CCT interventions with the goal to reduce the negative attentional vigilance bias that high-anxious individuals often display towards negative information, assuming that this will result in reduced anxiety. Based on the attentional control theory [24], anxiety is proposed to impair the efficient functioning of the goal-directed attentional control system, while at the same time increasing the extent to which cognitive processes are influenced by bottom-up, stimulus-driven attentional capture, particularly when stimuli carry threat-related or negative information. Anxious individuals with negative attentional vigilance bias detect and process more easily threatening stimuli, which distracts them away from task-relevant information and thus impacts the on-going task performance. Based on this theory, researchers have developed interventions - termed attention bias modification (ABM) - designed to train attention away from threatening stimuli with the expectation that this will lead to long-term reduction in vigilance to threat and in turn anxiety [41]. ABM typically uses an adaptation of the dot probe task with emotional words or faces. Unfortunately, despite earlier encouraging results, recent meta-analyses have demonstrated mixed evidence for reducing anxiety symptoms [42–45]. Findings from these meta-analyses highlighted that attention bias in anxiety is multifaceted and more dynamic than just a hypervigilance towards threat. As such, attention bias modification programs that simply train attention away from threat may too narrowly target one specific attentional vigilance mechanism that contributes to anxiety.

In the past years, there has also been growing interests in using video games for delivering interventions to help adolescents to learn to better regulate their emotions using non-stigmatizing games that have the potential to increase adherence to treatment [46–48]. A line of research within that field, interested in the impact of videogames on emotions, has documented the short-term beneficial effects of playing casual or puzzle videogames on stress, anxiety and mood [49–52]. Casual video games are video games targeted for a large audience, which are fun, fast to access, and simple to learn. They are also efficient at restricting attention to the game, possibly via the induction of Flow, acting as a powerful distraction from anxious thoughts and daily stress. In several interventions, Russoniello and colleagues have shown that participants assigned to play a casual game of their choice three times per week, for 30 min, over a 1-month period, showed substantial reduction in stress, anxiety and depression [49–52]. While these interesting effects on anxiety were assessed shortly after the intervention, whether their impact on emotion regulation and anxiety may be longer-lasting remains understudied.

### The multiple object tracking (MOT) paradigm and dual tasking

To better address the variety of attentional and regulation issues noted in anxious individuals, both at the bottom-up level and at the top-down level, we aimed at designing a training intervention that encourages the coordinated use of multiple attentional control functions at the same time– rather than focusing on a single attentional function. The Multiple Object Tracking (MOT) task is a paradigm that not only adequately reflects the demands of complex and dynamic human environments but also gathers several aspects of attentional control functions, including sustained and distributed attention as well as visuo-spatial working memory [53]. The MOT task consists of focusing attention on a limited number of moving targets appearing among moving distractors. After selective attention is applied to select a target set, sustained attention to the moving targets is in high demand as moving targets become indistinguishable from moving distractors. Such tracking requires both sustained and distributed attention as task-relevant stimuli move across the screen among other, as similarly looking distractors. Attentional demands vary depending on the number of targets to track, the speed of moving targets, as well as the distance between targets and distractors as tracking proceeds. In sum, tracking objects in a dynamic scene requires a demanding maintenance and updating of object information in working memory as well as sustained attention to the location of the to-be-tracked items [54, 55].

In the context of CCT, the MOT paradigm has been used to train cognitive functions with different populations (i.e. athletes, military, patients with neurodevelopmental disorders) and encouraging results have been reported. For example, Tullo [56] used a 3D MOT to train attentional abilities in adolescents (11–15 years old, *N* = 129) with neurodevelopmental disorders (i.e. autism spectrum disorder; attention deficit hyperactivity disorder, intellectual disability). Significant improvement of attention was observed in the MOT training group compared to both the distraction group (who played the game *2048)* or the passive no-training group. Harris et al. [57] did an intervention with 84 students randomized to a MOT training group or a passive no-training group. They showed that MOT training improved WM performance compared to the control condition. Vartanian et al. [58] conducted an intervention (*N* = 41 members of the Canadian Armed Forces) with MOT training that also showed improvements in WM performance in the training group compared to a passive no-training control group. Nyquist et al. [59] also used MOT training with low vision children and reported greater enhancement of their peripheral vision compared to a control video game training (*Tetris*), with improvements still noted 12 months after training.

To our knowledge, training attentional control skills through the MOT training regimen has never been investigated in anxious adolescents. Based on the link between impairments in attentional control abilities and anxiety highlighted in the literature, the MOT paradigm appears as a promising training regimen to target attentional control, and possibly reduce anxiety. We designed a training intervention based on the MOT paradigm described below and made it all the more demanding on attentional control by adding a visual detection task that automatically summon attention to the to-be-detected item. Such dual-task demands during training are known to facilitate attentional enhancement [60].

### Gamified training

Another feature of the proposed intervention is the inclusion of the MOT-detection dual task in a gamified environment. It has been proposed that intervention strategies which use gamification and computerized games have the potential to increase the adherence and impact of cognitive interventions [61]. We expect the implementation of game elements in the MOT-dual task training to be specifically suited for anxious adolescents for several reasons. Within this age range, there is a need for developing new digital interventions to support mental health and well-being. Video games are immersive and motivate a wide range of behaviors in adolescents, with 79% of Swiss youth playing video games every day on average one hour during the week and two hours during the weekend [62]. Similarly, 70.2% of Israeli teenagers play video games at least four times a week and spend on average two hours daily on this activity [63, 64]. As such, video games (both serious and entertainment video games) represent one of the most appealing digital interventions for addressing the issues of accessibility and engagement frequently encountered in mental health interventions [48, 65]. Video game elements can make this training intervention more enjoyable for adolescents, through the use of a familiar medium that feels safe, gratifying and immersive, thus helping adolescents to better maintain their motivation during an extensive training schedule [66].

In the context of mental health, a growing body of findings highlight the benefits (i.e., cognitive, motivational, and emotional) video games may confer upon users [48, 67–71]. For instance, by prescribing commercialized casual video games to adults, with the instruction to play three times per week, each session lasting thirty minutes, over a one-month period, a few randomized studies have established a causal reduction in stress, depression and anxiety [49, 51]. Scholten et al. [72] investigated the efficacy of a biofeedback video game for adolescents with high levels of anxiety compared to a distraction video game (*Rayman 2*: *The Great Escape*) and both conditions showed reduction in anxiety, which is promising for upcoming interventions studying video game effects on mental health.

### Study objectives

**Fig. 1:**
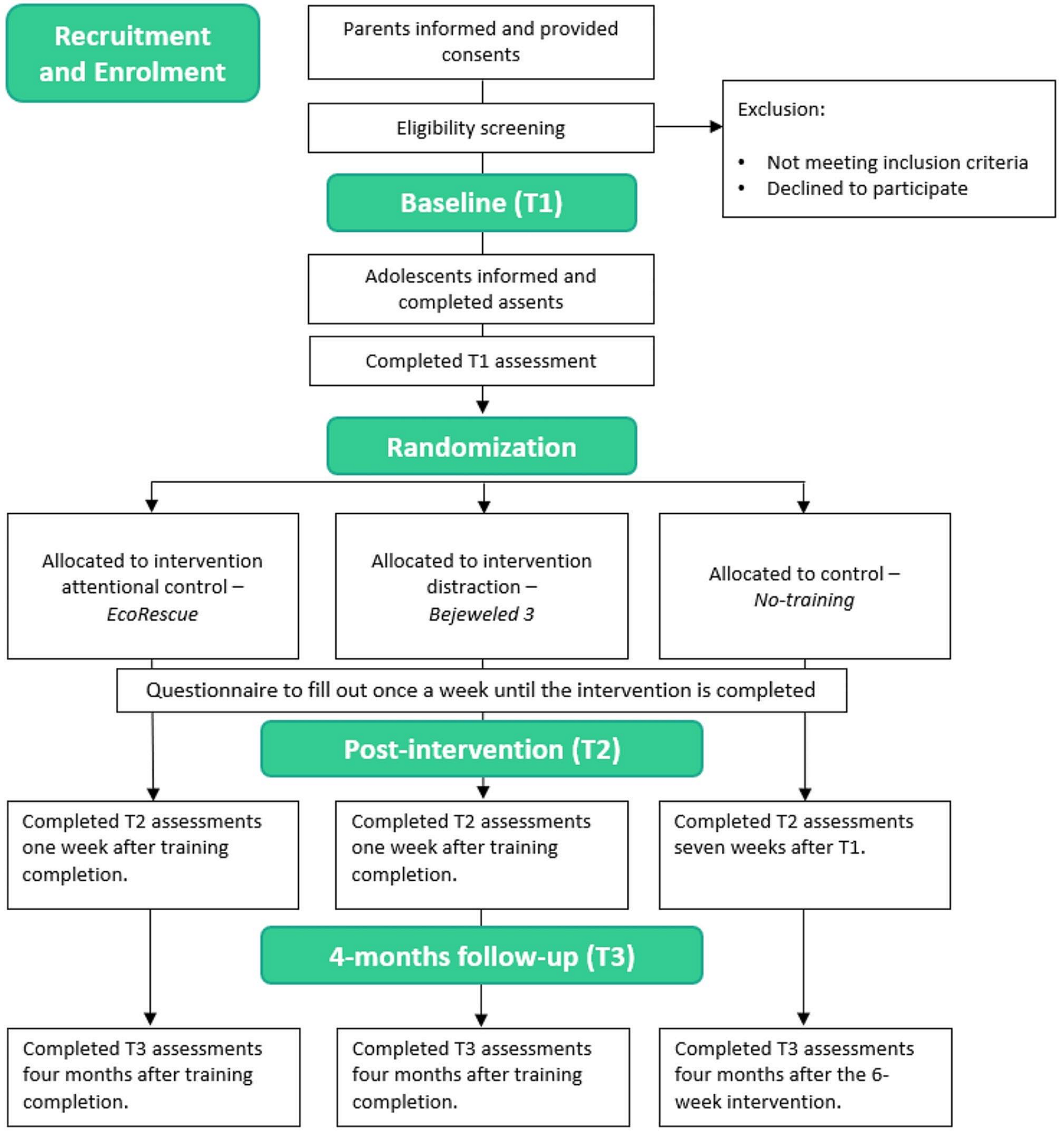
Flowchart of the proposed randomized controlled trial. Recruitment, screening, and randomization to training will be done online, while baseline, post and follow-up assessments will be administered on sites in either the Geneva or Haifa laboratories

The aim of this three-arm randomized controlled trial is to evaluate the feasibility of a 6-week video game training intervention to reduce adolescent anxiety-related symptoms. This study will also provide a first window into the efficacy of the proposed treatments when entirely deployed on-line in a young adolescent sample. The first group of participants will receive *Eco-Rescue*, an action game-based intervention (i.e. dual-task training) designed to train attention control. A second group will receive *Bejeweled 3*, a commercialized casual video game (i.e. puzzle game) that does not specifically target attentional control but has shown to reduce anxiety in previous studies, arguably through providing a distraction away from anxious thoughts [49]. A third, no-training control group will not receive any training, providing a needed control for any test-retest effects and allowing us to assess the extent to which the two video game genres proposed may impact self-report and behavioral outcomes (see Fig. 1 for study flowchart).

### Trial design and hypotheses

This randomized controlled trial study uses a multi-center, assessor-blind, superiority trial with three parallel group (3 groups × 3 time points) with primary and secondary endpoints assessed at baseline (T1), one week after completing the 6-week intervention or no-training period (T2) and 4 months after completing the assigned intervention (T3). We will investigate the following hypotheses:

#### Primary endpoint

*Video Game Intervention Hypothesis*. Anxiety may be reduced in adolescents through video game play because of their beneficial effects on attentional enhancement and/or the distraction away from anxious thoughts such play activity may trigger. We expect a significant effect on reducing anxiety symptoms from baseline (T1) to post-assessment (T2) for the two intervention groups (*Eco-Rescue*, *Bejeweled*) compared to the control group (*No-training*). Anxiety will be assessed with the Child Version of the self-report questionnaire of the Screen for Child Anxiety Related Emotional Disorders (SCARED-C).

#### Secondary endpoints

*Attentional Control Hypothesis*. Attentional control may be improved with an action game-based intervention. We expect the Eco-Rescue intervention to significantly increase attentional control from baseline (T1) to post-assessment (T2) compared to the No-training group and possibly Bejeweled, as measured by computerized tasks including the Useful Field of View (UFOV) and the Test of Variables of Attention (TOVA).

*Affective Control Hypothesis*. Affective control (i.e. attentional control applied in the presence of emotional distractors) can be improved with an action game-based intervention. We expect an increase in affective control for the Eco-Rescue game training group from baseline (T1) to post-assessment (T2) compared to the No-training one and possibly Bejeweled, as measured by the Affective Backward Digit Span (ABDS) task.

#### Exploratory endpoints

*Near-Transfer.* We expect the Eco-Rescue intervention to significantly increase performance on the MOT task from baseline (T1) to post-assessment (T2) compared to the No-training and the Bejeweled groups.

*Relationship between Attentional Control and Anxiety*. We expect greater attentional control to be associated with lower anxiety-related symptoms at baseline-assessment. If confirmed, we will then investigate the extent to which attentional control improvements (as measured by the UFOV and TOVA) may be associated with greater diminution in anxiety scores (SCARED Child version).

*Relationship between Attentional Control and Mental Health outcomes*. As the literature highlights a link between attentional control and mental health outcomes, we will also investigate whether greater attentional control (as measured by the UFOV and TOVA) is associated with greater affective control and emotion regulation abilities, and fewer self-reported mental health problems (e.g. depression). Affective control will be assessed using the Affective Backward Digit Span (ABDS) task while emotion regulation abilities will be assessed using the Cognitive Emotion Regulation Questionnaire and depression by the Patient Health Questionnaire (PHQ- 8).

*Relationship between Attentional Control and* in-game learning. We will evaluate whether better progression in the Eco-Rescue game may be related to higher attentional control (as measured by the UFOV and TOVA).

*Relationship between computerized tasks and self-report measures.* We will ask how task-based assessments relate to self-report measures for (i) attentional control (UFOV and TOVA versus Attentional Control Scale) and for (ii) emotional control (ABDS versus Cognitive Emotion Regulation Questionnaire).

*Motivation.* We will look at the impact of motivation to play the game (as measured by the Intrinsic Motivation Inventory) on intervention outcomes contrasting the Eco-Rescue and the Bejeweled groups.

*Expectation*. We will look at expectation about the intervention effects (based on the Expectation Questionnaire) contrasting the Eco-Rescue and the Bejeweled groups.

*Durability of effects*. We will explore long-term changes in intervention outcomes between baseline (T1) and follow-up (T3).

## Methods

### Study setting

This study will be completed both online and in the laboratory; more precisely, all game-related training will occur on-line, from the comfort of participants’ home, whereas all pre, post and follow-up assessments will be carried out in the laboratory. The study will be carried in parallel at two different sites: the Bavelier lab at the University of Geneva in Switzerland and the Shechner lab at the University of Haifa in Israel.

### Eligibility criteria

Eligible adolescents are those aged 12 to 14 years old, who own or have access to a computer, have access to the Internet, have an active mobile phone number, and score at 17 or higher on the parent version of the Screen for Child Anxiety Related Disorders (SCARED-P). Participants will be ineligible if they have a history of neurological injury (i.e. head injury), a diagnosed neurological or neurodevelopmental disorder (i.e. autism spectrum disorder), a diagnosed intellectual disability, a diagnosed psychosis or bipolar disorder or if they are currently enrolled in another cognitive training intervention. Uptake of other anxiety interventions during the study is permitted and will be assessed at screening, post and follow-up.

### Participants recruitment

150 adolescents will be recruited through flyers, advertisements on the lab website, mental health adolescent units, and social media platforms advertising the study in Geneva and Haifa. Study adverts will direct interested participants and their families to a Qualtrics page. There, they can review the study information, may choose to undertake screening and provide consent to be signed by the parent or legal guardian using the provided links. Regarding the recruitment site in Geneva, participants will be provided with contact information for local mental health and crisis support services in the information form to provide adequate information in case participants require care and support. Direct contact between the participant and the study team will not be required during recruitment and study enrolment. After signing the consent form, families will be redirected to online questionnaires to screen participants’ suitability for inclusion. Eligible participants will be redirected to a short contact form to collect their name, phone number and email address to be contacted by the research team and set up an appointment at the University of Geneva (Switzerland) or at the University of Haifa (Israel). Personal information will be stored in our secure system on university servers. Adolescents recruited will be provided with adapted study information and will sign an assent form before starting the baseline assessment. This study has been approved by the Ethics Board of the University of Geneva (CUREG) and by the Institutional Review Board (IRB) of the University of Haifa.

### Sample size

A power analysis indicates 26 participants per group are needed given a repeated measure ANOVA design looking at a 3-within (time of assessment) x 3–between (training conditions) interaction, Hedge’s G of -0.25, alpha level of 0.05 and power of 0.8, and a repeated-measure correlation of 0.70 (test-retest correlation on SCARED from Behrens et al., [73]; and Hedge’s G from the meta-analytic work of Wang et al. [74]. Our analyses design, however, calls for assessing differences between each of the training groups and the control group, or between the 2 training groups. For such 2 × 2 ANOVA design, with the same characteristics as above, 38 participants per intervention group is needed. We therefore plan on a total of 114 participants (38 for each of the three groups) in the final sample. Planning a 30% drop-out rate, we therefore expect to recruit 150 participants (50 for each of the three groups).

### Randomization and allocation

The study will have two training arms and one passive arm. After completing baseline assessment, participants will be randomized to one of the three following arms: the attentional control - Eco-Rescue intervention, the distraction - Bejeweled intervention or the No-training control group. Arm allocation will be concealed to the blinded experimental staff by using computer-generated condition assignment stratified by sex and anxiety score at baseline (assessed by the SCARED-C). To this end we will use the algo detailed in Sella et al. [75]. Such a procedure retains the value of a fully randomly assigned design, while simultaneously minimizing between group variance on the to be matched variables. Randomization will be performed separately for each site. Experimenters blinded to arm allocation will conduct assessments at each timepoint (baseline, post- and follow-up). The experimenters unblinded to the arm allocations will be responsible for administering the general instructions and technical support at any stage of the study and will take care of monitoring participants during the six weeks intervention period. Statistical analyses will be conducted by researchers blind to arm allocation.

### Interventions

#### Attention control training game - Eco-Rescue

*Eco-Rescue* is an action-like game designed and developed collaboratively by Swann Pichon, Naima Gradi and Daphné Bavelier at Campus Biotech Geneva. It is played from a third-person perspective on a PC or laptop. *In Eco-Rescue*, the player takes the role of a pilot tasked to depollute and reforest planets to restore communication and navigation between planets and galaxies. To accomplish this mission, the player will recruit residents of the planet displaying different emotions (i.e., happy, angry, or neutral) to depollute and catch butterflies that will help to reforest planets.

The mechanics of *Eco-Rescue* builds on the Multiple Object Tracking task [53] combined with a dual visual detection task. It requires players to divide, maintain and update the location of faces (with neutral, positive, or negative emotional valence) in visual working memory as they track the movement of different ships and their owners. Concurrently, a detection task requires players to press a key as soon as they detect the apparition of a butterfly to collect it. The stimuli of the detection task can only appear while the ships are moving. By requiring dual-tasking, its game play challenges task-switching abilities as well as the capacity to alternate between different states of attention (focused and distributed) as the game contingency changes, all the while requiring sustained attention.

A key feature of this game is difficulty scaffolding, which refers to the layering of increasingly complex levels of learning objectives in sync with players’ performance. Keeping players in such zone of proximal development has long been known to facilitate learning and video game design excels at doing so [76–78]. The MOT-detection task difficulty is adapted to each player’s performance by adjusting the speed at each number of to-be-tracked moving ships, whereas the difficulty of the detection task remains fixed. The MOT difficulty is adapted through a 3up-1down staircase algorithm programmed to automatically increase or decrease the speed of moving ships to track to maintain player’s performance around 80% accuracy [79, 80].

At each trial, the game provides immediate feedback on correct targets and performance. At the end of each level, the game provides delayed feedback on overall game performance and rewards earning. To avoid automatization of performance and reduce boredom, some variability has been built in. For the detection task, players cannot predict when and where the stimulus will appear on the screen. For the MOT task, the number of targets to track and the number of distractors varies from one trial to another to ensure varied task difficulty.

#### Distraction training game– Bejeweled 3

*Bejeweled 3* is a commercially available puzzle video game, developed and edited by PopCap Games. In this intervention, participants will play on their computer (PC or laptop) via Steam to match the physical setting of the Eco-Rescue attentional control intervention condition.

The main objective of *Bejeweled 3* is to swap adjacent colored gems to create a line or row of 3 or more gems of the same color. The gems so line-up disappear allowing the player to collect points. The goal is to get as many points as possible until it is impossible to swap the gems to line them up. *Bejeweled 3* includes different game modes players can discover freely at their own pace to promote a more enjoyable experience and variability during game sessions (one game usually lasts less than 5 min).

*Bejeweled 3* was chosen as it requires few attentional resources, especially when it comes to divided attention and switches between focused and divided attention (in contrast to *Eco-Rescue)* and because it was reported to reduce stress and anxiety in adult samples [49, 50].

### Outcomes

#### Primary outcome

*Anxiety.* Adolescent anxiety symptoms will be assessed using the Screen for Child Anxiety Related Disorders (SCARED-C [81]). SCARED-C is a 41 items self-report anxiety questionnaire assessing symptoms of Anxiety with subscales measuring General Anxiety Disorder, Social Phobia Disorder, Panic Disorder and Separation Anxiety Disorder. Severity of each symptom is rated referring to the past month on a scale ranging from 0 (Not True or Hardly Ever True) to 2 (Very True or Often True). The total SCARED score will be computed. Higher score indicates higher levels of anxiety.

#### Secondary outcomes

*Attentional control– far-transfer*. Attentional control will be assessed with the Useful Field of View (UFOV) task used by Joëssel [82] and an adaptation of the Test of Variables of Attention (TOVA) as implemented in the ACE-X battery. In the UFOV, participants have to identify whether a briefly flashed smiley presented at the center of the screen has short or long hair and detect on which of the eight cardinal directions was a peripheral target stimulus presented ignoring other distractors. The presentation time is made shorter (more difficult) or longer (less difficult) according to a 3-down-1-up staircase. The task stops after 8 reversals or 72 trials, whichever happens first. Performance is measured by the mean presentation time of the last 5 trials (expressed in milliseconds). This divided attention task measures the efficiency with which attention can be divided over the whole screen and attentional selection still proceeding in the context of distracting information. In the TOVA, participants have to press a response key when presented with a visual stimulus at the top of the screen (go trials) and refrain from responding if flashed at the bottom of the screen (no-go trials). A first block evaluates sustained attention and a second impulsivity. In the sustained attention, go trials represent 25% of the trials; in the impulsivity block, go trials represent 75% of the trials.

*Attentional control– near-transfer.* The Multiple Object Tracking task (MOT) will be used to assess near-transfer of training, given its use in the Eco-Rescue training. The MOT task is similar to the one used by Joëssel [82]. A total of 16 moving circles, including blue (targets) and yellow (distractors), will appear on the computer screen. Participants are presented with an array of yellow smiley faces moving freely within a circular area. During the first two seconds of each trial, a subset of these items is flagged as targets by being blue. The blue smileys then turn yellow and become indistinguishable from the other items. After four seconds, the items stop, one item is highlighted, and the participants have to respond whether this item was initially flagged blue or not. Participants complete a total of 65 trials, 5 trials with 1 target and 12 trials with 2 to 6 targets. MOT performance is measured as the percentage of correct responses (i.e. accuracy) in trials with 3, 4, 5 and 6 targets independently and combined overall. Greater accuracy score indicates better attentional control abilities.

*Affective working memory updating.* Affective working memory updating will be assessed with a modified version of the Affective Backward Digit Span task of Schweizer et al. [83]. Participants are presented with digits (1500ms/digit) in serial order presented on a background of either neutral or affective images to manipulate valence. The images are from the Geneva Affective Picture Database. In each trial, participants are presented with a series of single digits [0–9]. At the end of each trial, participants enter the series of digits seen in reverse order. No feedback is provided as to their answers. In the original version of the task, each span level is presented twice and at least one out of two correct trials per span level is needed for progression to the next level. If both trials are incorrect the task is terminated. In our version, the length of the series to remember is increased or decreased by one digit depending on previous trial success or failure according to a 1-up 1-down staircase algorithm. After two reversals the block stops. This is done once with neutral backgrounds and once with affective background images. Span is estimated for each block as the maximum correct length recalled. The span difference between the two contexts, neutral versus affective is an index of the individual ability to implement attentional control in the face of affective distraction (i.e. affective control).

#### Exploratory outcomes

*Attention Control Self-report*. The Attention Control Scale (ACS [84]) is a 20-item self-report questionnaire designed to measure the construct of attention focusing and of attention shifting. Participants rate items on a 4-point Likert Scale from 1 (almost never) to 4 (always) according to their experience in the past month.

*Emotion Regulation Self-report.* The Cognitive Emotion Regulation Questionnaire (CERQ [85]) will be administered to assess emotion regulation. The CERQ is a 36-item self-report measure that captures stable-dispositional cognitive emotion regulation strategies when people experience stressful or threatening life experiences. Specifically, the following strategies are measured: Self-blame, Blaming others, Acceptance, Refocusing on planning, Positive refocusing, Rumination, Positive reappraisal, Putting into perspective, and Catastrophizing. Participants rate items on a 5-point Likert Scale from 1 (almost never) to 5 (almost always) according to their experience in the past month.

*Depression Self-report*. Participants will complete the modified 8-item version of the Patient Health Questionnaire (PHQ-8 [86]) to assess symptoms of depression at each assessment time point of the study (T1, T2 and T3). Participants are asked to indicate how often they have experienced eight possible problems or symptoms over the last 2 weeks (e.g., “feeling down, depressed, or hopeless,” “feeling tired or having little energy,” and “feeling bad about yourself, or that you are a failure, or have let yourself or your family down”). Each item is rated on a four-item scale (0 - not at all, 1 - several days, 2 - more than half the days, or 3 - nearly every day). Items are summed to obtain scale scores.

*Sleep and Mood Self-report.* Sleep and Mood will be assessed through stand-alone questions for the duration of the training once a week. Sleep: “During the past week, how would you rate your sleep quality overall. This question is rated from 1 (very poor) to 5 (very good). Positive mood: “How much have you experienced positive mood in the past week?” This question is rated from 1 (not at all) to 5 (extremely). Negative mood: “How much have you experienced negative mood in the past week?” This question is rated from 1 (not at all) to 5 (extremely).

*Worry Self-report*. Worry will be assessed with the following statement taken from the Strengths and Difficulties Questionnaire (item 8 from the emotional problems scale [87]) for the duration of the training once a week. “During the past week I worried a lot.” This item is rated 0 (not true), 1 (somewhat true), or 2 (true).

*Intrinsic Motivation Self-report*. Intrinsic motivation for game play will be assessed just at the end of training for the intervention groups only (Eco-Rescue and Bejeweled) using an adapted version of the *Inventory Motivation inventory* (IMI [88]). We will use the same IMI version as in Joëssel [82], that is a 17-item self-report questionnaire with subscales measuring effort/importance, interest/enjoyment, perceived competence, perceived choice and pressure/tension. Participants rate each item on a 7-point Likert scale from 1 (not at all) to 7 (very true), higher score meaning higher motivation.

*Intervention Expectation Self-report*. Expectancy of training intervention on cognition, mood, efficiency and physical fitness will be assessed at post-test (T2) using an adaptation of the Expectation Questionnaire [89]. Participants will indicate on a 5-point Likert scale from 1 (totally disagree) to 5 (totally agree) to what extent the four following statements apply to them regarding their expectations about the study: “I think that participating in this study improved my [1. thinking skills (e.g., memory, attention, speed, reasoning) / 2. mood (e.g. more energetic, less stressed or anxious) / 3. ability to complete different tasks at school and at home (e.g., more efficient in accomplishing my tasks, less procrastination, better coordination with others) / 4. physical fitness].”

### Participant timeline

Participants will be enrolled in the study following eligibility screening, after they approved and signed the consent and assent forms. After completing the baseline assessment (T1) which will include computerized tasks and questionnaires, participants will be randomly assigned to one of the three arms: (1) attentional control - *Eco-Rescue*, (2) distraction - *Bejeweled*, or (3) control - *No-training*.

#### Training and control procedures

Participants in the attentional control (*Eco-Rescue*) and the distraction (*Bejeweled*) conditions will be asked to download the digital platform Steam on their computer to access their allocated games (see below for descriptions). We will ask participants to complete an expected total training time of 12 h, spread over 4 to 8 weeks, preferably distributed in 4 sessions of 30 min each per week for 6 weeks. Participants in the control (*No-training*) condition will live their life as usual during the intervention period. During the training period, every participant will be contacted by an unblinded researcher once a week to maintain contact and will be asked to fill out a self-report questionnaire about worry, sleep and mood. After completing the intervention, participants in the training groups will fill out a questionnaire about their motivation while playing the allocated game (Intrinsic Motivation Inventory -IMI).

Within one week and no less than 24 h after the end of the training phase, participants will complete the post- assessment (T2). Four months after completing their intervention, participants will complete the follow-up assessment (T3). Each assessment point (T1, T2, T3) will last around one hour and a half. Assessments will include as primary outcome the SCARED-C questionnaire, and as secondary outcomes distal and proximal measures of attention (UFOV task and TOVA for distal; MOT task for proximal), as well as a measure of affective control (Affective Backward Digit Span task). As for exploratory outcomes, assessments will include self-report measures of emotion regulation (CERQ), attention (ACS) and of depression (PHQ-8). At the end of follow-up assessment, all participants will fill out a questionnaire measuring their expectations toward the intervention.

### Adherence and retention

All participants will be compensated in gift cards for each section of the study to incentivize enrollment and study completion. They will receive 10 CHF (or 50 NIS) upon completing each of the baseline-, post- and follow-up assessments (T1, T2 & T3). All participants will be compensated 5 CHF (or 25 NIS) for completing the weekly surveys administered during the intervention phase. To maximize retention, all participants will be told that an additional bonus of 40 CHF (or 200 NIS) will be donated to a charity acting for climate change if they fully complete the study until the follow-up assessment. The two intervention groups (Eco-Rescue and Bejeweled) will be compensated 25 CHF or 175 NIS for completing the entire training.

Retention will be enhanced through weekly phone calls, during which participants will be requested to complete a brief survey. Participants in the training groups (Eco-Rescue and Bejeweled) will be updated on the training time remaining, while participants in the control group (No-training) will be updated on the time remaining before the next assessment.

### Data collection

We expect the study enrolment to begin in January 2024 and we project data collection to be finalized by June 2025 with a total recruitment of 150 participants.

### Data management

All collected data will be coded according to the ethical rules of the institutions collaborating to this project. Because we use secured servers located in Switzerland to acquire behavioural and questionnaire data for both sites, the data of both sites will be consolidated in Geneva. Only a single file on each research site will contain personal identifiable information (e.g., name, date of birth and email) and will be stored separately from other files and securely. Note that this file and the personal information it contains will not be shared between the two sites. This means that no personal information whatsoever will be exchanged between Geneva and Haifa. Personal information will only be used and accessed by the local researchers to contact participants recruited locally for the experiment.

### Risk assessment and monitoring

Whilst we do not expect the occurrence of adverse events or any particular risk to the health or safety of participants - beyond possible boredom or frustration during the activities– we will ask parents and adolescents to report every time an adverse event may occur with the intervention. The research team will be notified, and a decision will be made according to the severity of the adverse event. In case important protocol modifications are made they will be updated on the registration of the trial in clinicaltrials.gov.

### Data analysis

Primary analyses will be conducted on the entire randomized sample (i.e., intention to treat). The efficacy of our RCT trial will be established by considering change in anxiety (SCARED-C), between baseline (T1) and post-intervention (T2) assessments, based on the interaction between time and condition (the attentional control group compared to the no-training group and the distraction group compared to the no-training group), using parametric statistics, including mixed-effects repeated measure ANOVAs and t-tests. Missing data will be handled via full information maximum likelihood (FIML). FIML is a “gold standard” approach to handling missing data, assuming that data is missing at random (MAR). To examine whether this assumption may have been met, prior to running the primary analysis, we will examine patterns of missing data via pattern-mixture models. The LMM included fixed effects of time, group, and a group × time interaction, with random intercepts of subject and site, as well as a random slope of time. Statistical significance will be assessed at a 2-sided P-value of *P* <.05. We will apply a Bonferroni adjustment to correct for multiple comparisons.

Primary and secondary hypotheses will be tested through 2 (conditions - between subject factor) by 2 (time– within subject factor) repeated measure ANOVA’s. A significant group x time interaction in repeated measure ANOVAs will be interpreted as supporting our hypothesis. Main effects and significant interactions will be followed by post-hoc paired t-tests corrected for multiple comparisons.

Exploratory analyses will consist of parametric statistics, including t-tests and mixed repeated measure ANOVAs, simple correlations as well as mediation models to test our exploratory hypotheses (e.g. related to the relationship between attentional control and anxiety). Advances analytic methods will be developed to better address the question of whether the characteristics of learning in the Eco-Rescue gameplay progression may be related to changes in attentional control and anxiety, as there are no currently accepted methods to evaluate training related changes (e.g. in-game learning curves) nor to link them with performance gains (e.g. baseline-post cognitive changes) [90].

## Discussion

This study describes a 3-arm multi-site randomized controlled trial that aims to evaluate the effects of two potential beneficial paths for acting on anxiety using video games with adolescents. The first game, Eco-Rescue, is designed to be adaptive and to implement action-like video game mechanics to promote attentional control [69, 91–93]. The second video game, Bejeweled 3, is a casual video game previously used as a positive and relaxing distraction tool and shown to decrease anxiety, depression and stress in adults [49, 50]. We hypothesize that adolescents in the attentional control (Eco-Rescue) and in the distraction (Bejeweled) intervention arms will show significantly fewer anxiety symptoms at post-test than those in the control (No-training) arm. To our knowledge, this is the first study to evaluate the impact of action-like and casual video gameplay in an at-home intervention, with minimal personal guidance in adolescents. While video games are often considered as appealing and rewarding to the population [48], their potential to augment engagement and adherence rates of mental health interventions is yet under-documented. The publication of these results will be an occasion to further document how video game-based approaches to mental health may be best harness to address adolescents’ mental health issues.

Another strength of this study is to not only assess the impact of our two different types of video game play a few days after training completion, but also at 4 months follow-up. Indeed, we expect the Eco-Rescue action-like game to change for the better attentional control and this in a long-lasting fashion. A handful of studies indeed have documented that the positive impact of action video games is long lasting (months to year), whether on attentional control but also vision, mental rotation, or reading. In contrast, the impact of the Bejeweled 3, distraction game may be more fleeting. This study will also provide valuable insights about the expected duration of the effects depending on the mechanism of change targeted.

## Limitations

Although the proposed design includes a follow-up at 4 months, it is possible that some positive effects of the games on mental health and cognitive outcomes be even more protracted, taking 12 to 18 months to be visible. This is especially the case for outcomes relying on instruments such as self-report or everyday life performance, as illustrated in other literature [91, 94, 95].

Another limitation is that our implementation of action-like mechanics exploits the strength of the Multiple Object Tracking task within a closed loop system to its fullest, yet we recognize that this mechanic may become repetitive after 12 h of training. The proposed Eco-Rescue game would benefit from integrating other possible action-like game mechanics to increase gameplay variability and complexity [96, 97].

## Conclusion

Investigating the possibility of using video games to address mental health issues during adolescence is a promising route for the emerging field of digital mental health. This study will contribute to building up our knowledge of how to use video games to bring to this understudied population a form of help that is aligned with their daily activities and interests.

## Data Availability

The de-identified data generated and analyzed during the current study, will be made available through a data repository conform to FAIR regulations (OSF).

## Abbreviations

ABDS: Affective Backward Digit Span
ABM: attention bias modification
AC: attentional control
ACS: Attention Control Scale
CCT: computerized cognitive training
CERQ: Cognitive Emotion Regulation Questionnaire
CUREG: Ethics Board of the University of Geneva
IMI: Inventory Motivation inventory
IRB: Institutional Review Board
ITT: intention-to-treat
MOT: Multiple Object Tracking
UFOV: Useful Field of View
TOVA: Test of Variables of Attention
PHQ-8: Patient Health Questionnaire 8
RCT: randomized controlled trial
SCARED: Screen for Child Anxiety Related Disorders
WM: working memory
