# Correction: Evaluating the effect of action-like video game play and of casual video game play on anxiety in adolescents with elevated anxiety: protocol for a multi-center, parallel group, assessor-blind, randomized controlled trial

**DOI:** 10.1186/s12888-024-05669-4

**Published:** 2024-04-02

**Authors:** Naïma Gradi, Adrien Chopin, Daphné Bavelier, Tomer Shechner, Swann Pichon

**Affiliations:** 1 Department of Psychology, University of Geneva, Geneva, Switzerland; 2 Smith Kettlewell Eye Research Institute, San Francisco, CA USA; 3 Department of Psychology, University of Haifa, Haifa, Israel; 4 Geneva School of Health Sciences, Geneva, Switzerland

**Correction: BMC Psychiatry 24:56 (2024)**

**https://doi.org/10.1186/s12888-024-05515-7**

Following the publication of the original article [1], the authors would like to correct the text in the subheading Exploratory outcomes. The provided information was about the expectation questionnaire, this should have been about the adaptation to the adolescents. The updated changes have been highlighted in **bold typeface**.

The incorrect text is: *Intervention Expectation Self-report*. Expectancy of training intervention on cognition, mood, productivity and fitness will be assessed at 4 months follow-up (T3) using an adaptation of the Expectation Questionnaire [89]. Participants answer four questions rated on a 5-point Likert scale from 1 (unsuccessful) to 5 (successful): “How successful do you think playing Eco-Rescue/Bejeweled 3 has been at improving your [cognition (i.e. memory, attention, speed, reasoning) / mood (e.g. more energetic, less stressed or anxious) / productivity at work and/or at home (e.g. more efficient in completing my tasks, better at coordinating with others) / physical fitness]?

The correct text is: *Intervention Expectation Self-report*. Expectancy of training intervention on cognition, mood, **efficiency** and **physical** fitness will be assessed at post-test (T2) using an adaptation of the Expectation Questionnaire [89]. **Participants will indicate on a 5-point Likert scale from 1 (totally disagree) to 5 (totally agree) to what extent the four following statements apply to them regarding their expectations about the study: “I think that participating in this study improved my [1. thinking** skills (e.g., memory, attention, speed, reasoning) / 2. mood (e.g. more energetic, less stressed or anxious) / **3. ability to complete different tasks at school and** at home (e.g., more efficient in accomplishing my tasks**, less procrastination**, better coordination with others) / 4. physical fitness].

The original article [1] has been corrected.
